# Biochar and Intercropping With Potato–Onion Enhanced the Growth and Yield Advantages of Tomato by Regulating the Soil Properties, Nutrient Uptake, and Soil Microbial Community

**DOI:** 10.3389/fmicb.2021.695447

**Published:** 2021-08-27

**Authors:** Xingjia He, Hua Xie, Danmei Gao, M. Khashi U. Rahman, Xingang Zhou, Fengzhi Wu

**Affiliations:** ^1^Department of Horticulture, Northeast Agricultural University, Harbin, China; ^2^Key Laboratory of Cold Area Vegetable Biology, Northeast Agricultural University, Harbin, China

**Keywords:** microbial community, biochar, co-occurrence networks, tomato/potato–onion intercropping, bacteria, fungi

## Abstract

The application of biochar stimulates the activities of microorganisms that affect soil quality and plant growth. However, studies on the impacts of biochar mainly focus on a monoculture, its effects on interspecific interactions are rarely reported. Here, we investigated the impacts of biochar on tomato/potato–onion intercropped (TO) in a pot experiment. Tomato monoculture (T) and TO were treated with no, 0.3, 0.6, and 1.2% biochar concentrations in a pot experiment. Microbial communities from tomato rhizosphere soil were analyzed by quantitative PCR and Illumina MiSeq. The results showed that compared with the tomato monoculture, 0.6%TO and 1.2%TO significantly increased tomato yield in 2018. TO and 1.2%TO significantly increased plant height and dry weight in 2018 and 2019. Biochar treatments increased soil pH, decreased NO${}_{3}^{-}$-N and bulk density, and increased the absorption of N, P, and K by tomato. Bacterial and fungal abundances increased with an increase in biochar concentration, while *Bacillus* spp. and *Pseudomonas* spp. abundances showed an “increase-decrease-increase” trend. Biochar had a little effect on bacterial diversities but significantly lowered fungal diversities. TO, 0.6%TO, and 1.2%TO increased the potentially beneficial organisms (e.g., *Pseudeurotium* and *Solirubrobacter*) and lowered the potentially pathogenic organisms (e.g., *Kribbella* and *Ilyonectria*). Different concentrations of biochar affected the bacterial and fungal community structures. Redundancy analysis indicated that the bacterial community was strongly correlated with soil pH, NO${}_{3}^{-}$-N, and EC, while the fungal community was closely related to soil NO${}_{3}^{-}$-N and moisture. The network analysis showed that biochar and intercropping affected the symbiosis pattern of the microorganisms and increased the proportion of positive interactions and nitrifying microorganisms (Nitrospirae) in the microbial community. Overall, our results indicated that monoculture and intercropping with biochar improved soil physicochemical states and plant nutrient absorption, and regulated soil microbial communities, these were the main factors to promote tomato growth and increase tomato productivity.

## Introduction

Biochar refers to the organic material rich in carbon and stable in nature obtained from biomass pyrolysis at a low temperature under anaerobic or hypoxic conditions (Sun et al., 2016). It is often used as a soil amendment for sustainable agricultural purposes because of its porous structure, high surface area, and strong adsorption capacity. So far, the documented beneficial effects of adding biochar to soil include an increase in soil pH, soil structure improvement, increased nutrient content, and better crop yield (Gul and Whalen, 2016; Zheng et al., 2018). However, most of the previous studies focused on biochar amendment in monoculture systems (Liang et al., 2014; Gul et al., 2015), the effects of biochar addition in intercropping are barely evaluated. Therefore, understanding the impact of biochar on soil nutrients and crop yields in the intercropping system, which itself has proved vital for improving both soil and plant health (Li X. G. et al., 2018), could provide the basis for developing a strong strategy for sustainable agriculture.

Soil microorganisms are vital to agroecosystem functioning and sustainability (Zhou and Wu, 2018). The change in soil physical and chemical properties combined with plant growth can cause changes in the soil microenvironment, thus affecting soil microbial community composition (Zhu et al., 2017). Gomez et al. (2014) found that an increase in microbial abundance was positively correlated with the proportion of biochar application (0–20% mass). Similarly, Cheng et al. (2018) demonstrated that adding biochar altered the rhizosphere community and increased the relative abundances of *Adhaeribacter*, *Rhodoplanes*, and *Pseudoxanthomonas* in the soil. However, the response of microbial communities to crop diversification and, at the same time, soil-amended biochar has not been well explored yet, which could improve our understandings of the changes in microbial occurrence in response to multiple changes in the soil environment.

Studies have found that the surface absorbency of biochar affected the availability of soil nutrients (Biederman and Harpole, 2013; Zheng et al., 2018). Thangarajan et al. (2018) found that biochar reduced the loss of nitrogen and phosphorus in soil by absorbing and retaining NO${}_{3}^{-}$-N, NH${}_{4}^{+}$-N, and phosphate, and increased the availability of potassium to increase the uptake of K by crops. Besides, the application of biochar could also regulate nutrients availability by the changes in the abundance and diversity of soil microorganisms (Zhang Y. et al., 2018). For example, some beneficial organisms in the soil, such as *Sphingomonas* spp. and *Pseudomonas* spp. had been found promoting nutrient uptake by plants (Nwachukwu, 2001; Han et al., 2017). However, it is not clear whether the changes in microbial communities in response to biochar amendment in intercropping could affect nutrients availability in soil.

Intercropping refers to the effective agronomic practice of the simultaneous cultivation of two or more crops in the same area, which has been proved effectively utilizing natural resources, such as light, water, nutrients, heat, and land (Li et al., 2016; Liang et al., 2020). The increased crop yield by the enhanced nutrient availability and uptake and inhibition of soil-borne diseases in intercropping is closely related to the changes in the associated soil microorganisms (Bardgett and van der Putten, 2014; Zhang et al., 2021). Intercropping has also been shown to regulate the activation and uptake of N, P, and K and trace elements in rhizosphere soil (Inal et al., 2007). Thus, studying the combined impacts of intercropping and manually applied biochar on soil microbial community composition, nutrients availability, and overall soil physicochemical properties could provide us with novel insights into the biological functioning in the concealed belowground environment.

Soil microorganisms exist in complex interaction systems that determine the microbial community structure (Freilich et al., 2010). In recent years, the co-occurrence network analysis has become an effective means to explain the complex symbiotic relationships among soil microorganisms, providing new ideas for analyzing the interactions among soil microbial communities and promoting the understanding of the ecological niche space of community members (Dai et al., 2018). Previous studies have mostly focused on the association between soil microorganisms in biochar or intercropping single factors (Li and Wu, 2018; Asiloglu et al., 2021). However, little is known about the interaction between soil microorganisms in intercropping systems with biochar.

In our study, we investigated the effects of biochar on tomato growth, soil physicochemical properties, nutrient uptake, rhizosphere microbial community, and symbiosis pattern in the tomato monoculture (T) and tomato/potato–onion intercropping (TO) in continuous-tomato soil. The purpose of this study was to evaluate the improvement effect of biochar on continuous soil-associated culture and the interaction effect between biochar and intercropping. To this end, the soil physicochemical properties and tomato nutrient uptake were estimated by the Continuous Flow Analyser, and Quantitative PCR (qPCR) and Illumina MiSeq sequencing were used to analyze the abundance and composition of soil microbial community.

## Materials and Methods

### Site Description and Experimental Design

This study was conducted in the greenhouse of the Horticulture Experimental Station (45°41′N, 126°37′E) of Northeast Agricultural University in Harbin, China, from April 2018 to July 2019. The soil was black soil (Mollisol), where tomato has been continuously grown in a greenhouse for 10 years, and the texture was sandy loam soil. The soil physicochemical properties were as follows: pH 6.91 (1:2.5, w/v), EC 0.65 mS cm^–1^ (1:2.5, w/v), NH${}_{4}^{+}$-N 17.58 mg kg^–1^, NO${}_{3}^{-}$-N 180.38 mg kg^–1^, available P (AP) 98.68 mg kg^–1^, available K (AK) 240.00 mg kg^–1^, and bulk density 1.03 g cm^–3^. The biochar was prepared with corn stalk (preparation temperature was 450°C, provided by Shenyang Longtai Biological Engineering Co., Ltd., pH 8.43, EC 1.27 mS cm^–1^, NH${}_{4}^{+}$-N 8.61 mg kg^–1^, NO${}_{3}^{-}$-N 38.82 mg kg^–1^, available P 106.12 mg kg^–1^, available K 3,540.00 mg kg^–1^, bulk density 0.35 g cm^–3^). Tomato (*Solanum lycopersicum* L.) cultivar “Dongnong 708” and potato–onion (*Allium cepa* L. var. *aggregatum* G. Don) cultivar “Wangkui” were used in this study.

Pot experiment: biochar was evenly mixed with the tested soil at a mass rate with no, 0.3, 0.6, and 1.2% biochar, and plastic pots (24 cm × 22 cm) were filled with 5 kg of biochar amended soil in each pot during April 2018. Tomato seedlings with five leaves were transplanted to biochar amended soils after 3 days of the amendment as one seedling per pot. To set up the monoculture and intercropping treatments, one tomato was planted in one pot for the tomato monoculture, while three potato–onion bulbs were planted alongside tomato (6–10 cm) on the same day of tomato transplantation to the pots. The experiment was set up in a randomized complete block design with three replicates for each treatment (Supplementary Figure 1). Water was given regularly to keep the moisture about 55 ± 5% of the soil water holding capacity, weeds were removed manually, and no fertilizers were applied. For the second year of experiment, the tomato plants were transplanted on May 1, 2019, with the same planting method and field management as in 2018.

### Soil and Plant Sampling

After 30 days of tomato growth in the experimental pots, samples for various parameters were collected in 2018. The plant height was measured by meter sticks and leaves two ears of tomato to pick heart, and three strains were selected for each replicate to measure the yield in 2018. After 30 days of tomato growth in the experimental pots, three random tomato seedlings were harvested against each replicate for collections dry weight and nutrient uptake analysis as well as for the collection of tomato rhizosphere soil in 2019. The method of soil sample collection was according to Jin et al. (2020). Briefly, the tomato seedlings were uprooted carefully and shaken to remove loosely attached soil from the roots. Then, the rhizosphere soil was collected using a sterile brush to remove any soil tightly attached to the roots. The rhizosphere soils collected from all three seedlings in each replicate were pooled together, and each treatment had three replicates. Rhizosphere soil samples were stored at −80°C for DNA extraction. A soil sample collected from the tomato root zone was air-dried (<30°C) for soil physicochemical properties analysis.

Harvested tomato seedlings were washed, and the moisture from the root surface was absorbed by absorbent paper; finally, the tomato seedlings were dried at 105°C in the oven for 30 min, and then dried to a constant weight in the oven at 75°C. The dry weight of the tomato seedlings was measured in an electric weight balance, then the plants were crushed, sieved through a 0.3 mm mesh, and the plant nutrient concentration and uptake were analyzed.

### Determination of Growth Indicators and Nutrients in Tomatoes

The dried and crushed plant samples of 0.10 g were cooked with H_2_SO_4_–H_2_O_2_ at 380°C. Discoloration liquor was stored at 4°C, and the concentrations of N, P, and K in plants were determined by the Continuous Flow Analyser (SAN++, Skalar, Breda, Netherlands), and nutrient uptake was calculated using the following formula:

Nutrientuptake=⁢Nutrientconcentrations⁢×Dryweightoftheplant

### Determination of Soil Physicochemical Properties

Soil physicochemical characteristics were analyzed by the method described by Bao (2005). Soil pH and EC were determined in a soil-water suspension (1:2.5, w/v) using a glass electrode and conductivity meter, respectively. For soil inorganic nitrogen concentration (NH${}_{4}^{+}$-N and NO${}_{3}^{-}$-N), soil available P and K were extracted with 2 M KCl, 0.5 M NH${}_{4}^{+}$OAc (pH = 7.0) and 1 M NaHCO_3_ (pH = 8.5), respectively, and then soil filtrates were determined by the Continuous Flow Analyser (SAN++, Skalar, Breda, Netherlands). Soil moisture contents were determined by drying the soil at 80°C in the oven to a constant weight. Soil bulk density was determined by the cutting ring method (Liu et al., 2020).

### DNA Extraction and Quantitative PCR

Soil total DNA was extracted from 0.25 g of soil of each replicate using the MoBioPowerSoil^TM^ DNA Isolation Kit (Mo Bio Laboratories Inc. Carlsbad, CA, United States) according to the instructions of the manufacturer. Electrophoresis in a 1.2% (w/v) agarose gel stained with ethidium bromide was performed in order to check the yield and quality of the extractions. Each composite soil sample was extracted in triplicate and the extracted DNA solutions were pooled. There were three composite DNA solution samples for each treatment. The DNA concentration and purity were determined with a NanoDrop 2000 Spectrophotometer (Thermo Scientific, Waltham, MA, United States).

Quantification of 16S rRNA and ITS genes were performed on an iQ5 Real-Time PCR Detection System (Bio-Rad Lab, Hercules, CA, United States). For the bacterial communities, the primer was 338F/518R (Muyzer et al., 1993), and each 20 μL of PCR reaction contained 9 μL of 2 × Real SYBR Mixture, 0.2 μL of each primer (10 μM), 2.5 μL of template DNA, and 8.1 μL of ddH_2_O. The qPCR reaction conditions were as follows: 5 min at 95°C for initial denaturation, 22 amplification cycles of 50 s at 95°C for denaturation, 30 s at 65°C for annealing, 1 min at 72°C for an extension, 10 min at 72°C for a final extension, and the amplified fragment length was about 230 bp. For the fungal communities, the primer was ITS1F/ITS4 (Gardes and Bruns, 1993), and each 20 μL of the PCR volume contained 9 μL of 2 × Real SYBR Mixture, 0.2 μL of each primer (10 μM), 2.5 μL of template DNA, and 8.1 μL of ddH_2_O. The qPCR reaction conditions were as follows: 5 min at 94°C for initial denaturation, 28 amplification cycles of 1 min at 94°C for denaturation, 1 min at 63°C for annealing, 45 s at 72°C for an extension, 10 min at 72°C for a final extension, and the amplified fragment length was about 750 bp. For the *Bacillus* communities, the primer was BacF/BacR (Garbeva et al., 2003), and each 20 μL of the PCR volume contained 9 μL of 2 × Real SYBR Mixture, 0.3 μL of each primer (10 μM), 2.0 μL of template DNA, and 8.4 μL of ddH_2_O. The qPCR reaction conditions were as follows: 5 min at 94°C for initial denaturation, 28 amplification cycles of 1 min at 94°C for denaturation, 90 s at 57.4°C for annealing, 90 s at 72°C for an extension, 10 min at 72°C for a final extension, and the amplified fragment length was about 995 bp. For the *Pseudomonas* communities, the primer was PSF/PSR (Widmer et al., 1998), and each 20 μL of the PCR volume contained 9 μL of 2 × Real SYBR Mixture, 0.4 μL of each primer (10 μM), 2.0 μL of template DNA, and 8.2 μL of ddH_2_O. The qPCR reaction conditions were as follows: 5 min at 94°C for initial denaturation, 33 amplification cycles of 1 min at 94°C for denaturation, 1 min at 57.6°C for annealing, 2 min at 72°C for an extension, 10 min at 72°C for a final extension, and the amplified fragment length was about 960 bp. The standard samples were diluted to yield a series of 10-fold concentrations and subsequently used for the qPCR standard curves. The *R*^2^-value for each standard curve exceeded 0.99, indicating linear relationships over the concentration ranges used in this study. All of the amplifications were run in triplicate with the DNA extracted from each soil sample.

### Illumina MiSeq Sequencing and Data Analysis

The V4-V5 hypervariable region of the 16S rRNA gene and the ITS1 region of the fungal ITS gene were used as the bacterial-specific fragment and the fungal-specific fragment with the primer sets 338F/806R (Xu et al., 2016) and ITS1F/ITS2 (Bellemain et al., 2010), respectively. These primer pairs were modified with a 6-bp unique barcode sequence at the 5′ end to identify samples. All amplification was performed in 25 μL of reactions contained 0.5 μL of each primer, 1 μL of template DNA (10 ng), 2 μL 2.5 mM dNTPs, 0.5 μL of FastPfu Polymerase (Transgen Biotech, Beijing, China), 0.5 μL of ×5 FastPfu buffer, and 20 μL of ddH_2_O. The PCR conditions, performed in an ABI GeneAmp 9700 PCR System (ABI, Waltham, MA, United States), were as follows: 3 min of initial denaturation step at 95°C, followed by 35 cycles of 94°C for 30 s, 50°C for 30 s, and 72°C for 30 s, and a final extension step at 72°C for 10 min for the 16S V4-V5 rRNA gene and 3 min of initial denaturation step at 94°C, followed by 35 cycles of 94°C at 30 s, 55°C for 30 s, and 72°C for 45 s, and a final extension at 72°C for 10 min for ITS genes. The products from the three replicates amplification of the bacterial 16S rRNA gene, and fungal ITS gene were separately pooled and evaluated on 2% agarose gels (TBE buffer). Amplicons were purified with a DNA gel extraction kit (Axygen, China), quantified with a QuantiFluorTM-ST fluorometer (Promega, Madison, WI, United States), pooled at equimolar concentrations, and finally sequenced on an Illumina Miseq PE300 platform at Majorbio Bio-Pharm Technology Co., Ltd. (Shanghai, China), each treatment was done in triplicate.

The raw data were filtered and processed by using the QIIME software (Version 1.9.1) (Caporaso et al., 2010). The chimeras were discarded by using the “chimera.uchime” command in Mothur. The UPARSE (Version 7.0.1090) pipeline was performed for taxonomic assignment with similarities >97% (Edgar, 2013). Taxonomic classification was conducted with the GreenGenes (Version 135) and UNITE (Version 8.0) databases for bacteria and fungi, respectively. To preclude bias due to several sequencing depths, all samples were subsequently subsampled based on the minimum number of soil microbial sequencing depths of this study. The original data of bacteria and fungi in SRA is SRP277738.

In this study, the community composition of each sample was counted with the data of sub-sampled based on the minimum number at phylum and genus level, respectively. They have analyzed the changes of microbial abundance in different treatments at different levels. That is, at the phylum level, the dominant flora of bacteria (relative abundance > 5% in at least one treatment) and fungi (relative abundance > 6% in at least one treatment) were presented in a histogram. At the genus level, we analyzed the top 50 taxa, which can be classified and detected. Besides, the co-occurrence network diagram showed the changes of soil microbial communities at bacterial (relative abundance > 0.3% in at least one treatment) and fungal (relative abundance > 0.1% in at least one treatment) genus level.

### Statistical Analysis

The original test data were analyzed by Turkey’s HSD test in the SAS 9.2 software at a *p* < 0.05 level. The bar diagram was prepared using OriginPro 9.0. The α-diversity index of bacteria and fungi communities were calculated by the QIIME software (Version 1.9.1) (Caporaso et al., 2010), including Chao1 index, Shannon index, Inverse Simpson index, and Coverage index. The community structures of bacteria and fungi were visualized by Non-metric multidimensional scaling (NMDS) with Bray-Curtis dissimilarity matrices to clarify the differences in the community compositions of bacteria and fungi in the different treatments. Anosim and adonis were used to compare the microbial community differences of other treatments with the Bray-Curtis distance and 999 permutations. Mantel test and redundancy analysis (RDA) based on the euclidean distance were used to evaluate the relationship between bacterial and fungal community structures and physical and chemical factors. Based on strong *(p* > 0.8) and significant correlations (*p* < 0.01), the symbiotic patterns of soil microorganisms in different treatments were visualized by Gephi (Version 0.9.2). NMDS, ANOSIM, adonis, Mantel test, and RDA analyses were performed using the “vegan” package in “R” (Version 3.3.1, R Foundation for Statistical Computing, Vienna, Austria).

## Results

### Effects of Biochar Amendment and Intercropping on the Growth and Development of Tomato

**FIGURE 1:**
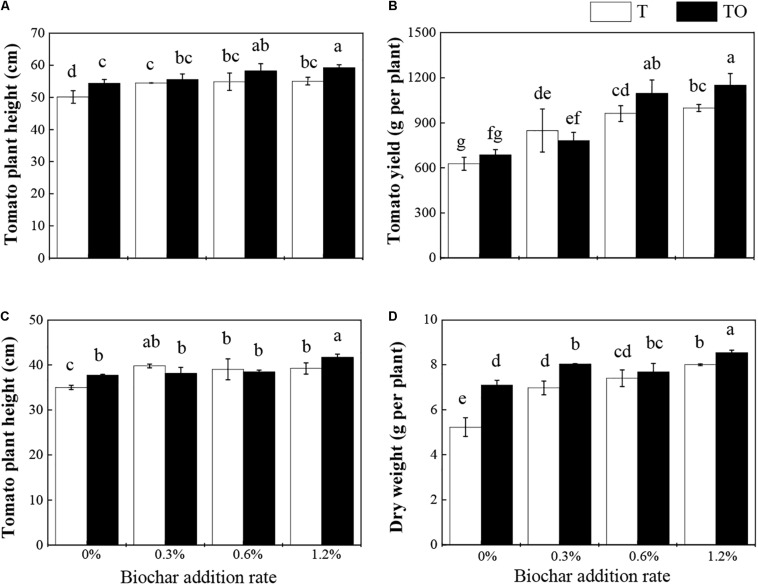
Effects of biochar and intercropping on tomato height **(A)** and yield **(B)** in 2018, tomato height **(C)** and dry weight **(D)** in 2019. Tomato monoculture (T) and tomato/potato–onion intercropping (TO) indicate tomato monoculture and intercropped with potato–onion, respectively. 0, 0.3, 0.6, and 1.2% indicate the biochar rates. Different letters indicate the significant differences (*P* < 0.05).

Compared with the tomato monoculture, intercropping with no biochar and monoculture and intercropping systems with biochar significantly increased the plant height of tomatoes. Among them, the intercropping system with 1.2% biochar was significantly higher than the monoculture system with 1.2% biochar in 2018 (*P* < 0.05; Figure 1A). Under the 0.6 and 1.2% biochar treatments, the yield of the intercropping system was significantly higher than their monoculture systems and the monoculture and intercropping systems with no biochar in 2018 (*P* < 0.05; Figure 1B). The intercropping system with no biochar and monoculture system with biochar significantly increased the plant height and dry weight of tomato (*P* < 0.05). Among them, the plant height and dry weight of the intercropping system with no biochar and 1.2% biochar were significantly higher than their monoculture systems in 2019 (*P* < 0.05; Figures 1C,D). These results indicated that intercropping and addition of biochar could improve the plant height, dry weight, and yield of tomato, and the intercropping with the addition of 1.2% biochar had the best effect.

### Effects of Biochar Amendment and Intercropping on the Soil Physicochemical Properties

Compared with the tomato monoculture, the intercropping system with no biochar significantly increased soil moisture content and pH (*P* < 0.05) but significantly decreased NO${}_{3}^{-}$-N and AK content (*P* < 0.05). Intercropping systems with 0.6 and 1.2% biochar significantly increased soil pH (*P* < 0.05), and intercropping system with 1.2% biochar significantly decreased soil bulk density and NO${}_{3}^{-}$-N content (*P* < 0.05; Table 1). The results indicated that intercropping and adding biochar had significant effects on the physical and chemical properties of the soil. The impacts of intercropping systems with no biochar and 1.2% biochar were more prominent.

**TABLE 1:** Effects of biochar and intercropping on the physicochemical properties of continuous-cropping tomato soil after 30 d pot experiment in 2019.

Treatment		pH	Moisture (%)	Bulk density (g⋅cm^–3^)	EC (mS⋅cm^–1^)	NH${}_{4}^{+}$-N (mg/kg)	NO${}_{3}^{-}$-N (mg/kg)	AP^a^ (mg/kg)	AK^a^ (mg/kg)
0%	T	6.84 ± 0.04d	20.32 ± 0.24b	1.0084 ± 0.0028a	0.45 ± 0.02abc	18.44 ± 0.70c	37.18 ± 0.23b	98.53 ± 3.74c	169.67 ± 1.70a
	TO	6.96 ± 0.04c	21.49 ± 0.47a	1.0042 ± 0.0027a	0.48 ± 0.01a	20.14 ± 0.31abc	17.82 ± 0.38f	103.19 ± 4.19bc	160.67 ± 7.59bc
0.3%	T	6.89 ± 0.05d	21.32 ± 0.71ab	1.0022 ± 0.0057a	0.39 ± 0.07bcd	23.14 ± 0.34a	42.41 ± 1.04a	103.91 ± 0.94abc	170.33 ± 1.70a
	TO	7.0 ± 0.06bc	21.58 ± 0.59a	1.0050 ± 0.0051a	0.39 ± 0.03cd	21.82 ± 2.18ab	12.94 ± 0.94g	109.11 ± 5.71ab	163.33 ± 2.05abc
0.6%	T	6.98 ± 0.03c	21.66 ± 1.03a	1.0079 ± 0.0055a	0.36 ± 0.02d	18.90 ± 1.36bc	22.60 ± 0.79d	103.33 ± 2.17abc	156.33 ± 2.05c
	TO	7.05 ± 0.02ab	21.23 ± 0.37ab	1.0109 ± 0.0086a	0.45 ± 0.05ab	18.46 ± 1.93c	19.58 ± 0.60e	110.11 ± 2.26a	158.67 ± 2.87c
1.2%	T	6.97 ± 0.01c	21.81 ± 0.88a	0.9748 ± 0.0097b	0.43 ± 0.01abc	20.57 ± 0.24abc	27.78 ± 0.28c	103.99 ± 1.04abc	168.33 ± 3.86ab
	TO	7.10 ± 0.04a	21.75 ± 0.28a	0.9427 ± 0.0148c	0.43 ± 0.01abc	21.01 ± 2.63abc	23.25 ± 0.28d	110.09 ± 3.07ab	162.67 ± 4.11abc

*T and TO indicate tomato monoculture and intercropped with potato–onion, respectively. 0, 0.3, 0.6, and 1.2% indicate the biochar rates. Different letters indicate the statistically significant differences among treatments (*P* < 0.05).*

*^*a*^AP and AK indicated soil available P and available K, respectively.*

### Effects of Biochar Amendment and Intercropping on Nutrient Uptake of the Tomato Plant

**FIGURE 2:**
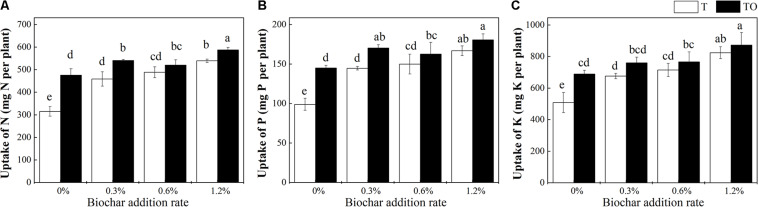
Effects of biochar and intercropping on uptake of N **(A)**, P **(B)**, and K **(C)** of tomato plant after 30 days pot experiment in 2019. T and TO indicate tomato monoculture and intercropped with potato–onion, respectively. 0, 0.3, 0.6, and 1.2% indicate the biochar rates. Different letters indicate the statistically significant differences among treatments (*P* < 0.05).

Compared with tomato monoculture, the intercropping system with no biochar and the monoculture and intercropping systems with biochar significantly increased the uptake of N, P, and K by tomato plant (*P* < 0.05; Figure 2). Among all the treatments, 1.2% biochar treatment had the most pronounced effect on improving the absorption of N, P, and K in the tomato plant. Compared with the tomato monoculture with no biochar, the N, P, and K of the intercropping system with 1.2% biochar were increased by 86.51, 82.60, and 71.50%, respectively. The N, P, and K of the monoculture system with 1.2% biochar were increased by 71.22, 68.65, and 62.02%, respectively.

### Soil Microbial Community Abundances

**FIGURE 3:**
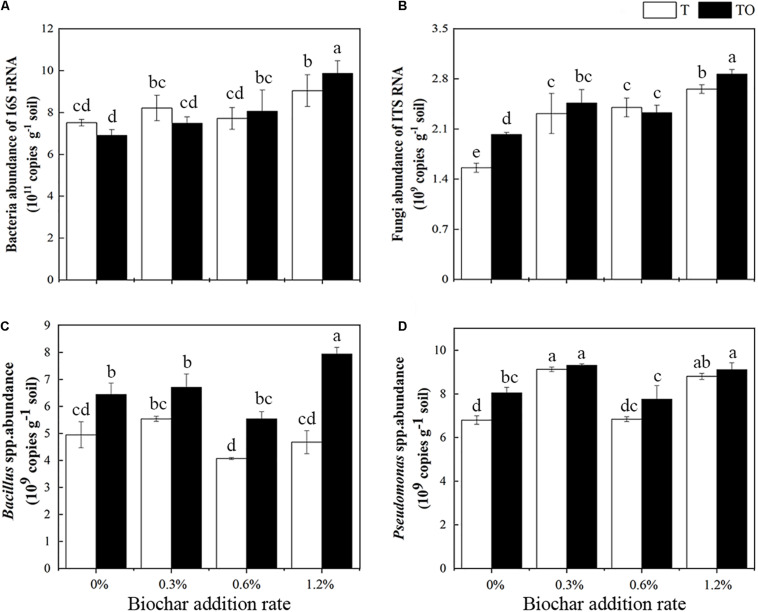
Effects of different concentrations of biochar amendment and intercropping on the abundances of total bacteria **(A)**, fungi **(B)**, *Bacillus* spp. **(C)**, and *Pseudomonas* spp. **(D)** in tomato rhizosphere soil after 30 days pot experiment in 2019. T and TO indicate tomato monoculture and intercropped with potato–onion, respectively. 0, 0.3, 0.6, and 1.2% indicate the biochar rates. Different letters indicate the statistically significant differences among treatments (*P* < 0.05).

We found that the abundance of total bacteria increased with an increase in biochar amendment in intercropping tomato, and it was highest in tomato intercropping with potato–onion with 1.2% biochar amendment (Figure 3A). Similarly, the abundance of total fungi was lowest in monoculture tomato followed by intercropping tomato, both with no biochar amendment, as compared to biochar amendments (*P* < 0.05). No differences were found in the monoculture or intercropping systems with 0.3 and 0.6% biochar amendment (Figure 3B). The abundance of *Bacillus* spp. in the intercropping treatments with no biochar and biochar was significantly higher than the monoculture, respectively (*P* < 0.05), and the abundance of *Bacillus* spp. in the intercropping system with 1.2% biochar was significantly higher than other treatments (*P* < 0.05; Figure 3C). Similarly, compared with the tomato monoculture, except for the monoculture with 0.6% biochar, the different treatments significantly increased the abundance of *Pseudomonas* spp., especially the intercropping and monoculture systems with 0.3 and 1.2% biochar (Figure 3D). These results indicated that the abundances of bacteria and fungi increased with the increase of biochar concentration, while the abundance of *Bacillus* spp. and *Pseudomonas* spp. showed an “increase-decrease-increase” trend. Among all treatments, adding 1.2% biochar had the most pronounced impacts on increasing soil microbial abundances.

### Bacterial and Fungal Community Diversities

The process of raw sequencing reads generated about a total of 1,295,820 high-quality V4–V5 sequences and 1,611,566 high-quality ITS1 sequences. The average read length of bacteria and fungi were 415 and 233 bp, respectively, while the average sequence per sample of bacteria and fungi were 53,993 and 67,149, respectively, based on a 97% identity threshold. The diversity was calculated with the minimum sequence number (45,192 for bacteria and 43,043 for fungi). In this study, the coverage of bacterial and fungal communities reached 95 and 99%, respectively (Table 2), which represented the true conditions of the total bacteria and fungi in the samples. For the bacterial communities, compared with the monoculture with no biochar, 0.3% biochar treatment significantly increased the Inverse Simpson index (*P* < 0.05), other treatments significantly decreased the Inverse Simpson index (*P* < 0.05). Monoculture with no biochar and monoculture and intercropping with biochar significantly decreased the Chao 1 index (*P* < 0.05). For the fungal communities, compared with the monoculture and intercropping with no biochar, 0.3, 0.6, and 1.2% biochar treatments significantly decreased the No. of OTUs and Chao 1 index (*P* < 0.05). In addition, 0.3 and 1.2% biochar treatments significantly decreased Shannon index and Inverse Simpson index (*P* < 0.05; Table 2). These results indicated that biochar had a little effect on bacterial community diversities but significantly reduced fungal community diversities.

**TABLE 2:** Diversity indices of soil microbial communities based on 16S rRNA and ITS genes were analyzed from the Illumina MiSeq sequencing at a 97% sequence similarity.

Classify	Biochar rate	Coverage (%)		No. of OTUs		Shannon		Inverse Simpson		Chao 1	
		T	TO	T	TO	T	TO	T	TO	T	TO
	0%	95.06	95.34	3,929 ± 108a	3,683 ± 123a	7.14 ± 0.06a	7.0 ± 0.13a	506.69 ± 44.17b	402.84 ± 125.51e	5,562 ± 97a	5,280 ± 130b
Bacteria	0.3%	95.13	95.27	3,830 ± 64a	3,816 ± 133a	7.02 ± 0.07a	7.09 ± 0.13a	372.38 ± 102.48g	509.86 ± 107.45a	5,431 ± 41ab	5,326 ± 174b
	0.6%	95.06	95.61	3,829 ± 57a	3,736 ± 171a	7.09 ± 0.05a	7.03 ± 0.01a	474.24 ± 77.43d	388.08 ± 6.96f	5,591 ± 77a	5,016 ± 64c
	1.2%	95.35	95.32	3,739 ± 66a	3,776 ± 28a	7.09 ± 0.05a	7.07 ± 0.03a	483.77 ± 38.05c	402.51 ± 19.70e	5,258 ± 118b	5,279 ± 37b
	0%	99.77	99.78	570 ± 29a	566 ± 14a	3.47 ± 0.03bc	3.62 ± 0.07a	11.14 ± 0.74a	12.75 ± 1.01a	801 ± 39a	756 ± 34a
Fungi	0.3%	99.74	99.82	532 ± 7b	482 ± 6d	3.43 ± 0.07cd	3.15 ± 0.06e	10.25 ± 0.59bc	7.71 ± 0.88c	665 ± 24b	651 ± 10b
	0.6%	99.81	99.84	526 ± 17bc	529 ± 24bc	3.55 ± 0.06ab	3.50 ± 0.06bc	11.73 ± 1.30a	11.40 ± 0.88a	693 ± 30b	646 ± 46b
	1.2%	99.80	99.82	530 ± 10bc	499 ± 27cd	3.41 ± 0.01d	3.33 ± 0.12d	10.28 ± 0.02bc	9.82 ± 1.84b	698 ± 18b	682 ± 50b

*T and TO indicate tomato monoculture and intercropped with potato–onion, respectively. 0, 0.3, 0.6, and 1.2% indicate the biochar rates. Different letters indicate the statistically significant differences among treatments (*P* < 0.05).*

### Soil Bacterial and Fungal Community Composition and Structures

**FIGURE 4:**
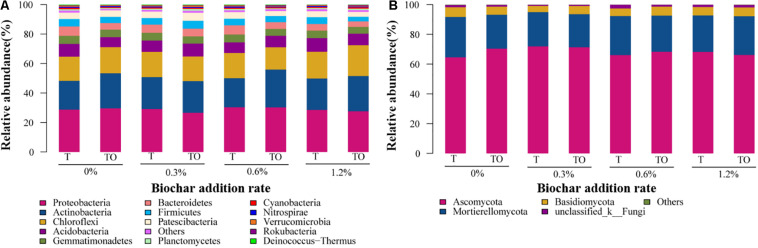
The relative abundances of bacteria **(A)** and fungi **(B)** phylum on tomato rhizosphere soil after 30 days pot experiment in 2019. T and TO indicate tomato monoculture and intercropped with potato–onion, respectively. 0, 0.3, 0.6, and 1.2% indicate the biochar rates.

The MiSeq sequencing data were classified at a 97% similarity level, and the phyla of 37 bacteria and 13 fungi were obtained. The predominant phyla of the bacterial community were Proteobacteria, Actinobacteria, Chloroflexi, Acidobacteria, Bacteroidetes, Gemmatimonadetes, Firmicutes, and Patescibacteria (relative abundance > 5% in at least one treatment), these phyla occupied more than 90% of the total sequences (Figure 4A). Moreover, unclassified bacteria accounted for 0.24–0.63% (Supplementary Table 1). The relative abundance of Actinobacteria was significantly higher with 0.6% biochar treatment, and Firmicutes was significantly lower with no and 1.2% biochar treatments in the intercropping system than the monoculture system (Figure 4A). Ascomycota, Mortierellomycota, and Basidiomycota were the dominant fungal phyla (relative abundance > 6% in at least one treatment) at all treatments, which accounted for more than 95% of the sequences (Figure 4B). Moreover, 0.77–2.48% of fungi were not classified (Supplementary Table 2).

**FIGURE 5:**
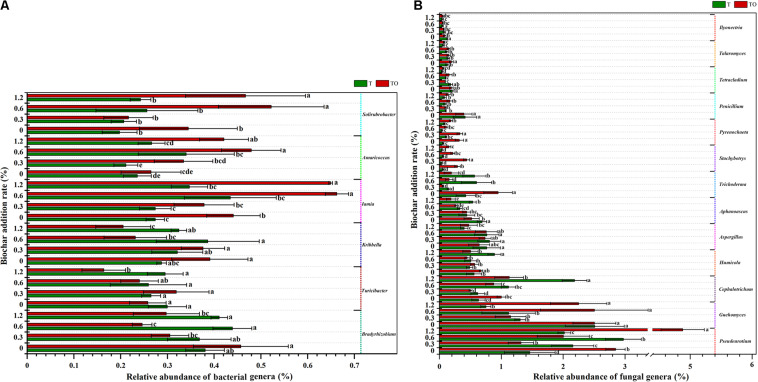
The relative abundances of bacterial genera **(A)** and fungal genera **(B)** in tomato rhizosphere soil changed significantly in the top 50 after 30 days pot experiment in 2019. T and TO indicate tomato monoculture and intercropped with potato–onion, respectively. 0, 0.3, 0.6, and 1.2 indicate the biochar rates (%) on the Y-axis. Different letters indicate the statistically significant differences among treatments (*P* < 0.05).

A total of 1,139 bacterial genera and 306 fungal genera were detected at the genus level. Supplementary Tables 3, 4 demonstrated the influence of the top 50 abundant classified genera of soil bacterial and fungal communities. We analyzed the bacterial and fungal genera with significant changes in the relative abundances (Figure 5). For the bacterial communities, compared with the tomato monoculture, intercropping significantly increased the relative abundances of *Iamia* and *Solirubrobacte*r with no biochar treatment, and *Amaricoccus*, *Iamia*, and *Solirubrobacter* with 0.6 and 1.2% biochar treatments (*P* < 0.05) but significantly decreased the relative abundances of *Bradyrhizobium* and *Kribbella* with 0.6 and 1.2% biochar treatments and *Turicibacter* with 1.2% biochar treatment (*P* < 0.05). Besides, the relative abundances of *Bradyrhizobium*, *Turicibacter*, and *Kribbella* with 1.2% biochar treatment were significantly lower and the relative abundances of *Iamia*, *Amaricoccus*, and *Solirubrobacter* with 0.6 and 1.2% biochar treatments were significantly higher in intercropping than no biochar (*P* < 0.05; Figure 5A).

For the fungal communities, compared with the tomato monoculture, intercropping significantly increased the relative abundances of *Pseudeurotium*, *Talaromyces*, *Trichoderma*, *Stachybotrys*, and *Pyrenochaeta* (*P* < 0.05) and significantly decreased the relative abundances of *Aphanoascus* and *Ilyonectria* with no biochar treatment (*P* < 0.05). The intercropping with 1.2% biochar treatment significantly increased the relative abundances of *Pseudeurotium*, *Guehomyces*, *Stachybotrys*, and *Pyrenochaeta* (*P* < 0.05) but significantly decreased the relative abundances of *Aphanoascus*, *Cephalotrichum*, *Humicola*, and *Trichoderma* (*P* < 0.05). Besides, the relative abundances of *Pseudeurotium* in the intercropping and monoculture systems with 1.2% biochar and the relative abundances of *Cephalotrichum* and *Humicola* in the monoculture with 1.2% biochar treatment were significantly higher than the monoculture (*P* < 0.05). The relative abundances of *Aphanoascus*, *Penicillium*, *Tetracladium*, and *Talaromyces* in the intercropping and monoculture with 1.2% biochar treatments; *Aspergillus*, *Ilyonectria*, and *Guehomyces* in the monoculture; and *Trichoderma*, *Stachybotrys*, and *Pyrenochaeta* in the intercropping system with 1.2% biochar treatment were significantly lower than no biochar (*P* < 0.05; Figure 5B).

**FIGURE 6:**
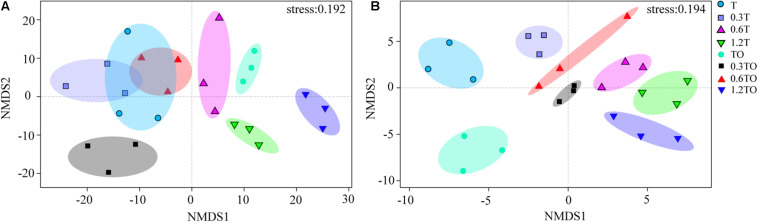
Non-metric multidimensional scale (NMDS) analysis of bacterial **(A)** and fungal **(B)** community structures based on Bray-Curtis distances at the OTU level. T and TO indicate tomato monoculture and intercropped with potato–onion, respectively. 0, 0.3, 0.6, and 1.2% indicate the biochar rates.

Non-metric multidimensional scaling analysis at the OTU level showed that the differences between bacterial and fungal β-diversity based on the Bray-Curtis distances dissimilarity (Figure 6). The results showed that the three repetitions of each treatment were clustered together, indicating that the bacterial community structure samples in this study had good repetitions. For the bacterial communities, there was no separation between the monoculture and monoculture with 0.3% biochar. There was no separation between the monoculture and intercropping with 0.6% biochar. The other treatments were clearly separated, indicating the obvious differences in the bacterial community structures between other treatments (ANOSIM, *R* = 0.950, *P* = 0.001; adonis, *R*^2^ = 0.541, *P* = 0.001; Figure 6A). For the fungal communities, the obvious separation of each treatment indicated that there were obvious differences in the fungal community structures between all treatments (ANOSIM, *R* = 0.988, *P* = 0.001; adonis, *R*^2^ = 0.676, *P* = 0.001; Figure 6B). These results indicated that the addition of biochar affected the community structures of bacteria and fungi in the monoculture and intercropping systems.

### Relationships Between Soil Microbial Communities and Soil Physicochemical Properties

**FIGURE 7:**
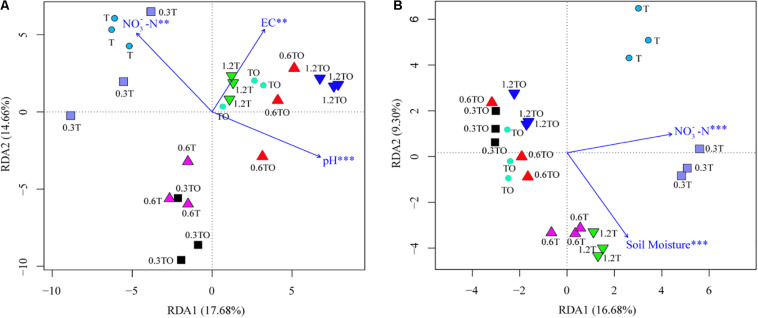
Redundancy analysis (RDA) of tomato rhizosphere total bacterial **(A)** and fungal **(B)** community structures at 30 days in 2019. The environment variables with statistical significance are presented by arrows. T and TO indicate tomato monoculture and intercropped with potato–onion, respectively. 0, 0.3, 0.6, and 1.2% indicate the biochar rates. **P < 0.01; ***P < 0.001.

Mantel test results showed that the change in bacterial community structure was associated with soil pH (*r* = 0.146, *p* = 0.044), EC (*r* = 0.164, *p* = 0.014), and NO${}_{3}^{-}$-N (*r* = 0.169, *p* = 0.024), while the fungal community structure was associated with soil NO${}_{3}^{-}$-N (*r* = 0.162, *p* = 0.045) and moisture content (*r* = 0.160, *p* = 0.013). RDA analyzed the relationship between the changes of the bacterial and fungal community structures and environmental factors among the different treatments (Figure 7). Soil pH (*r*^2^ = 0.41, *p* = 0.004), EC (*r*^2^ = 0.72, *p* = 0.001), and NO${}_{3}^{-}$-N (*r*^2^ = 0.40, *p* = 0.006) were the main factors driving the change of soil bacterial community structure (Figure 7A). Soil NO${}_{3}^{-}$-N (*r*^2^ = 0.68, *p* = 0.001) and moisture content (*r*^2^ = 0.59, *p* = 0.001) were the main factors driving the change of soil fungal community structure (Figure 7B).

### Co-occurrence Network Analysis

**FIGURE 8:**
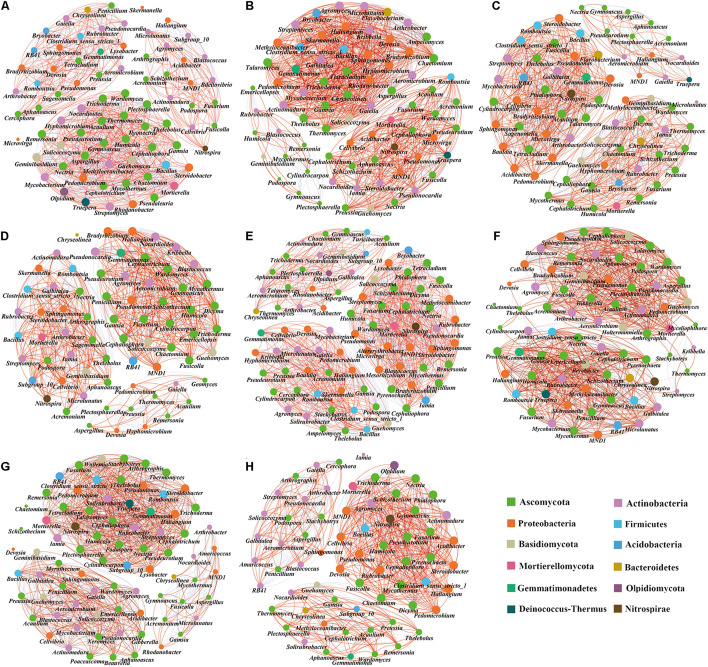
Co-occurrence network analysis of soil microbial communities in different treatments. **(A)** T, **(B)** 0.3%T, **(C)** 0.6%T, **(D)** 1.2%T, **(E)** TO, **(F)** 0.3%TO, **(G)** 0.6%TO, and **(H)** 1.2%TO. A link represents a significant correlation (Spearman, |ρ| > 0.8, *P* < 0.05). The red line indicates a positive correlation, and the blue line indicates a negative correlation. The node size is proportional to the number of connections. Only bacteria with genus level abundance >0.3% and fungi with genus level abundance >0.1% are shown. T and TO indicate tomato monoculture and intercropped with potato–onion, respectively. 0, 0.3, 0.6, and 1.2% indicate the biochar rates.

We compared the symbiotic relationships and community complexity of the bacterial and fungal communities in the different treatments using network graphs (Figure 8). The topological network properties of the different microbial communities are shown in Supplementary Table 5. The results showed that the modularity index of each treatment was greater than 0.4, indicating that the symbiosis network of each treatment was a typical modular structure. Compared with the tomato monoculture, the monoculture system with 0.3% biochar, intercropping systems with no, 0.3, and 0.6% biochar increased the total edge numbers of microbial networks (Figures 8E,B,F,G), while the monoculture systems with 0.6 and 1.2% biochar and intercropping system with 1.2% biochar decreased the total edge numbers of microbial networks (Figures 8C,D,H). The monoculture system with 0.3% biochar and the intercropping systems with 0.3 and 1.2% biochar increased the proportion of positive correlation sides in the total sides but decreased the proportion of negative correlation sides in the total sides (Figures 8B,F,H). Compared with the tomato monoculture, the microbial community network of intercropping was relatively complex (Figure 8E). In contrast, the microbial community networks of the monoculture and intercropping systems with 1.2% biochar were relatively simple (Figures 8D,H). In the tomato monoculture system, the pathogenic fungi *Aspergillus* and *Ilyonectria* were at the center of the network. They were closely related to other bacterial genera (Figure 8A), while the pathogenic fungi *Ilyonectria* did not appear in the monoculture and intercropping systems with biochar (Figures 8B–H). Intercropping and monoculture with biochar increased the proportion of nitrifying microorganisms (Nitrospirae phylum accounted for the proportion of the entire network phylum: T, 1.33%; 0.3%T, 1.40%; 0.6%T, 1.43%; 1.2%T, 1.47%; TO, 1.20%; 0.3%TO, 1.41%; 0.6%TO, 1.39%; 1.2%TO, 1.54%) in the whole network. Besides, the relationships between beneficial bacteria (e.g., *Pseudomonas* and *Bacillus*) and beneficial fungi (e.g., *Pseudeurotium* and *Humicola*) and nitrifying microorganisms (*Nitrospira*) were closer in the intercropping with 1.2% biochar (Figure 8H). These results indicated that the intercropping and monoculture systems with biochar simplified the relationship networks between bacteria and fungi, increased the positive interaction of microbial communities and enhanced the connection of beneficial organisms in the networks, and weakened the connection of pathogenic organisms in the networks.

## Discussion

### Effects of Biochar and Intercropping on the Soil Physicochemical Properties

The addition of biochar is an effective means to improve the physical and chemical properties of the soil and nutrient absorption, which has a significant effect on improving the quality of the soil environment (Li Y. F. et al., 2018; He et al., 2020). We found that the amendment of 1.2% biochar increased the soil pH and reduced the bulk density (Table 1), which is consistent with the findings of Biederman and Harpole (2013). Biochar is generally alkaline, and the change in soil pH may be attributed to the direct effect of biochar amendment (Yao et al., 2017). The bulk density of biochar used in this test (0.35 g/cm^3^) was much lower than that of the soil (1.03 g/cm^3^). Biochar addition generally reduced soil bulk density by 3–31%, reducing the consistency of the soil, indicating that bulk density decreased with biochar application, and better soil permeability was more conducive to plant growth (Omondi et al., 2016). Simultaneously, the effects of biochar on pH and bulk density were more advantageous in the intercropping and could be the reason for the increased pH (Cong et al., 2015) and decreased bulk density by intercropping (Gong et al., 2019). Besides, biochar application can change the nitrogen content in the soil. In northern China, nitrate nitrogen is the primary nitrogen in the soil, and its content directly reflects the short-term nitrogen supply in soil (He et al., 2018). We found that the NO${}_{3}^{-}$-N of intercropping was lower than monoculture, which indicated that the interaction between biochar and intercropping may be beneficial to the N uptake by plants.

### Effects of Biochar and Intercropping on Plant Growth and Nutrient Uptake of Tomato

Previous studies have shown that biochar can improve soil fertility, promote plant growth, and increase yield by providing soil nutrients (Steiner, 2008; Zheng et al., 2018). Moreover, the application of biochar regulated the biological resource supply to plants (N, P, and K) and improved the uptake of N, P, and K (Zhang Q. et al., 2019). Our study found that biochar of 1.2% increased the dry weight, yield, and uptake of N, P, and K in tomato plants of intercropping and monoculture (Figures 1, 2). The reason that biochar can promote the nutrient absorption of tomato plants may be that in the process of high-temperature decomposition of raw materials, organic phosphorus will be transformed into inorganic phosphorus, and a large amount of inorganic phosphorus will be retained in the biochar. The addition of biochar can also improve the C/N ratio in soil, thus improving the ability of the plant to absorb nitrogen and other nutrients. Therefore, the biochar provided enough nutrients to plant by acting as a “slow-release nutrient source” (e.g., N and P) (Steiner, 2008). Thangarajan et al. (2018) also suggested that biochar has a positive effect on plant growth and nutrient uptake. Besides, the interaction between biochar and intercropping may have a positive impact on the nitrogen cycle by regulating nitrogen mineralization and nitrification in soil (Marzaioli et al., 2018). All these results indicated that the application of biochar changed the content of organic matter in soil and increased the nutrients needed for crop growth. Moreover, the increase of plant diversity and the pore structure of biochar also provide a good environment for the growth and reproduction of soil microorganisms and maintain the nutrient cycle of the soil ecosystem. Simultaneously, intercropping and biochar can reduce the accumulation of autotoxic substances and reduce the harm of toxic elements (Brooker et al., 2015; He et al., 2020).

The improvement of plant nutrition supply, soil properties, and soil environment were the important reasons for biochar to improve crop dry weight and yield (Zwieten et al., 2010). Besides, the change of soil pH caused by biochar may change the acidic soil into medium alkaline soil, which may affect the forms of nutrient elements (e.g., P) and make it easier for plants to use (Ding et al., 2016). Our study also found that the dry weight and yield of the monoculture with 1.2% biochar were significantly higher than intercropping with no biochar, which indicated that the effect of the reasonable biochar addition on tomato productivity was greater than intercropping. This means that the reasonable combination of intercropping and biochar can bring the best benefits to crop productivity. This may be because intercropping itself can activate the insoluble nutrients in the soil (Li X. et al., 2020), while the unique properties of biochar can absorb nutrients, reduce the leaching of nutrients, and retain the availability of nutrients in the intercropping soil (Kuo et al., 2020). The combined effect of the two can maximize the growth promotion benefits. This result was confirmed in previous studies; Liu et al. (2017) showed that biochar significantly improved the yield advantage and relative nitrogen and phosphorus absorption advantage of maize/soybean and maize/peanut intercropping systems, and biochar enhanced the nitrogen fixation of legumes, thus expanding the benefits of legumes intercropping.

### Effects of Biochar and Intercropping on Tomato Rhizosphere Soil Microbial Communities

In recent years, the response of soil microorganisms to biochar has attracted much attention. The chemical properties (especially pH value and nutrient content) and physical properties (e.g., pore size, pore-volume, and specific surface area) of biochar played a critical role in determining the effectiveness of biochar on microbial performance because biochar provided a suitable habitat for microorganisms (Palansooriya et al., 2019). It was known from several studies that the amendment of biochar can increase the abundances of bacteria, fungi, *Bacillus* spp., and *Pseudomonas* spp. (Moore et al., 2018; Wang et al., 2019). Similarly, we found that biochar of 1.2% increased the abundances of bacteria, fungi, *Bacillus* spp., and *Pseudomonas* spp. (Figure 3). Pietikäinen et al. (2000) found that biochar has a high surface area, which can harbor bacteria, increasing their abundances. Yao et al. (2017) observed that biochar increased the copy number of fungal genes in the soil. The increase of the abundances of bacteria, fungi, *Bacillus* spp., and *Pseudomonas* spp. could also be indirectly caused by the change of soil physical and chemical properties (e.g., pH and bulk density) caused by biochar amendments (Ameloot et al., 2014). Besides, we also found that the abundances of bacteria and fungi increased with the increase of the concentration of added biochar change in the monoculture and intercropping systems. This was because of the change of the microbial abundances about applying biochar content. The results were also confirmed in the previous studies. For example, the increase of the biochar content from 0.5 to 5.0% (w/w) obviously changed the abundance of bacteria in the soil (Wang et al., 2015).

The study of microbial diversity is of great significance to the sustainable development of agriculture. Our study showed that biochar had a little effect on the bacterial community diversity. However, it decreased the fungal community diversity (Table 2). This may be because the impact of biochar on the microbial community largely depends on the application amount of biochar, biochar, and soil types. Microbial community diversity changed only when biochar application was high enough to significantly change the soil water holding capacity, pH conditions, and nutrient concentrations. These were the most critical factors affecting microbial diversity and soil composition (Zhang Y. et al., 2018). Moreover, Nguyen et al. (2018) also reported that the impact of biochar on the bacterial diversity was highly time-dependent, and biochar can temporarily improve the bacterial diversity but had a little impact on the bacterial community over time. In our experiment, one crop was planted in 2018. The high-throughput analysis was carried out 30 days after planting in 2019. This may be one of the reasons for the no significant change in the bacterial diversity in our study. Similar to our results, Hu et al. (2014) demonstrated that biochar amendment could reduce the fungal diversity. The results of the different effects of biochar on the diversities of bacteria and fungi may be because the bacteria were more likely to utilize nutrients and mineral elements through adsorption to the surface of biochar or colonization in the pores of biochar. The results indicated that the effects of biochar on soil microbial diversities were limited. They largely depended on the concentration of biochar added and the time of its retention in soil.

Soil microbial community relative abundance has been changed by applying biochar in the soil (Zhu et al., 2017; Cheng et al., 2018). We found that the addition of biochar had no significant effect on the main bacterial and fungal phyla but it affected the level of bacterial and fungal genus (Figure 4). Compared with the tomato monoculture, 1.2% biochar treatment had a lower relative abundance of *Bradyrhizobium*, *Kribbella*, and *Turicibacter* spp. but a higher relative abundance of *Solirubrobacter*, *Iamia*, and *Amaricoccus* spp. (Figure 5A). The results of previous studies showed that the *Kribbella flavida* was a type of pathogenic strain “Nocardioides Fulvus” IFO 14399 (Park et al., 1999). *Bradyrhizobium* rhizobium was involved in the nitrogen cycle (Anderson et al., 2011). The application of biochar may reduce the abundance of slow-growing rhizobia because it reduced the utilization of nitrogen sources by slow-growing rhizobacteria (Khan et al., 2014). Moreover, *Solirubrobacter* spp. had a positive impact on plant growth (Franke-Whittle et al., 2015). The results showed that biochar had a positive impact on increasing the relative abundance of potentially beneficial organisms (*Solirubrobacter* and *Pseudeurotium* spp.) and decreasing the potentially pathogenic organisms (*Kribbella* spp.). Previous studies reported that pathogens were inhibited by *Pseudeurotium* spp. (Heo et al., 2019). *Cephalotrichum* produced ectokeratase closely related to K (Gradisar et al., 2000). *Humicola* had the potential for ethylation (Chen et al., 2017a). We found that the treatments of no and 1.2% biochar significantly increased the relative abundance of *Pseudeurotium* spp. in intercropping than monoculture. As compared with no biochar, 1.2% biochar treatment also significantly increased the relative abundance of *Pseudeurotium* spp. in intercropping and monoculture and *Cephalotrichum* and *Humicola* spp. in monoculture (Figure 5B). This indicated that 1.2% biochar could improve the relative abundance of potentially beneficial fungi. Tramycin produced by *Aspergillus* spp. can cause death (Ferrari et al., 2017). *Ilyonectria* spp. was associated with fungal soil-borne diseases (Morales-Cruz et al., 2018). The results indicated that intercropping decreased the relative abundance of *Ilyonectria* spp. than monoculture with no biochar, and lowered the relative abundance of *Aspergillus* spp. by 1.2% biochar treatment and *Ilyonectria* spp. by 0.3, 0.6, and 1.2% biochar treatments in the monoculture system. This showed that biochar had diverse effects on the functional fungi in the soil, which increased the abundance of potentially beneficial fungi, and on the other hand, reduced the abundance of potentially pathogenic fungi. However, its impacts on more functional fungi need to be studied further. Moreover, tomato intercropped with potato–onion had an essential impact on increasing beneficial organisms, reducing pathogenic organisms, and promoting plant health (Li N. H. et al., 2020). When the suitable concentration of biochar was added, the beneficial organisms of monoculture increased significantly and the pathogenic organisms decreased significantly, especially in the intercropping systems (Zhang M. M. et al., 2018, 2019). This indicated that the interaction between intercropping and adding appropriate biochar had an ideal impact on improving soil health and promoting plant growth. However, only two intercropping crops with different concentrations of biochar were selected in this study. In the future, more crops with specific functions should be chosen to better study the diversity and composition of soil microbial communities under different planting systems and different concentrations of biochar.

The changed soil physical and chemical properties caused by biochar can affect the microbial community structure (Li Y. C. et al., 2018). Biochar itself has a large porosity, which can provide habitat conditions for the survival of microorganisms and protect them from predators (Warnock et al., 2007). The improvement of soil properties, such as soil pH, soil water retention capacity, and increased availability of nutrients (e.g., C, N, P, and K) can promote the growth of soil microorganisms and regulate the microbial community structure (Li et al., 2019). Non-metric multidimensional scaling analysis showed that the addition of biochar affected the community structures of bacteria and fungi in the different treatments (Figure 6). Further RDA showed that soil NO${}_{3}^{-}$-N, pH, and EC were the main factors influencing the bacterial community structure. Soil NO${}_{3}^{-}$-N and moisture content were significantly positively correlated with the fungal community structure (Figure 7). Studies have shown that the change of the bacterial community structure was closely related to the change of pH and EC caused by biochar (Chen et al., 2017b; Cheng et al., 2019), which may be related to the high pH in biochar and the improvement of soil properties required for a microbial colonization by EC. Our results also prove this point. Besides, the NO${}_{3}^{-}$-N varied greatly in our study, and the community structures of bacteria and fungi were positively correlated with NO${}_{3}^{-}$-N. This indicated that soil nitrogen resources might be critical environmental factors affecting soil microbial community structures. This is consistent with the results of Zainudin et al. (2020), who indicated that changes in soil nitrogen levels following the addition of biochar were the major factors affecting the soil microbial communities. However, the influences of soil characteristics on the community structure are different in different studies. For example, Zhang et al. (2016) pointed out that soil pH, organic carbon, and total nitrogen were the main factors affecting the bacterial community structure. Yao et al. (2017) also pointed out that NO${}_{3}^{-}$-N, pH, and moisture content were closely related to the changes of fungal community structure.

The complex interaction between species is one of the crucial indicators of community biodiversity (Dai et al., 2018). Intercropping with biochar significantly affected the symbiotic relationships between soil bacteria and fungi. Intercropping with no biochar increased the scale of the microbial co-occurrence network but decreased the proportion of positive correlation among communities. Monoculture with 0.6 and 1.2% biochar and intercropping with 1.2% biochar decreased the scale of the microbial symbiotic network but increased the proportion of positive correlation among communities (Supplementary Table 5). In the tomato monoculture system, the pathogenic fungi *Aspergillus* and *Ilyonectria* were at the center of the network and they were closely related to other bacterial genera (Figure 8A). However, the pathogenic fungi *Ilyonectria* did not appear in the monoculture and intercropping systems with biochar (Figures 8B–H). This indicated more co-existence (positive interaction) of microorganisms than mutual exclusion (negative interaction) in the treatments with biochar, which was more conducive to the stability of the whole microbial community. Studies have shown that to maintain the stability of the whole system, the more pathogenic microorganisms in the soil microbial community, the more complex the relationships between microorganisms were required, predator-prey interactions increased the complexity of the network (Allesina and Tang, 2012). However, the beneficial microorganisms (e.g., *Pseudomonas*, *Bacillus*, *Pseudoeutium*, and *Humicola*) in the intercropping with biochar, especially in 1.2% biochar, were in the critical position. At the same time, the fungal genus of *Ilyonectria*, which was related to soil-borne diseases, was not detected. This indicated that the pathogenic fungus genus of *Ilyonectria* was very sensitive to the intercropping and biochar treatments. The intercropping with biochar reduced the chance of infection by pathogenic microorganisms. This is similar to the research of Asiloglu et al. (2021), who suggested that biochar treatment can affect the presence of specific groups of microorganisms in the entire network and this effect was related to their specific physicochemical properties. The intercropping system with biochar can improve the positive relationships between the microbial communities, which may reduce the energy consumed by the whole microbial community to deal with the negative effects. This enhanced the resistance of the whole network and drove the whole network to develop in a more healthy direction. This view was also confirmed by Bello et al. (2020). Besides, the proportion of nitrifying microorganisms in the whole network increased with the addition of biochar, which was closely related to the transformation of nitrogen in the soil (Lücker et al., 2010). This may be one of the critical reasons for increasing nitrogen uptake by plants in the intercropping systems with biochar. Besides, compared with a tomato monoculture, intercropping increases plant diversity, resulting in different root exudates between monoculture and intercropping systems (Li N. H. et al., 2020). In the intercropping system, the amount and diversity of available carbon sources of soil microorganisms increased due to the increase of species diversity, which led to the changes of the amount, diversity, activity, and fecundity of soil microorganisms (Zhang M. M. et al., 2018). Root exudates are the media of communication between the plants and the external environment, which affect the availability of plant nutrients, and also constitute the main reason of different plant rhizosphere micro-ecological characteristics. Studies have shown that root exudates can affect soil microbial activity and interaction (Philippot et al., 2013; Li et al., 2017). There were significant differences in root exudates between monocropping and intercropping, which may be one of the reasons for the differences in the microbial interactions among other treatments.

## Conclusion

Overall, our results provided an empirical evidence that biochar application in monoculture and intercropping systems can improve soil physical and chemical properties, promote crop nutrient uptake, and improve crop productivity. Biochar and intercropping can increase the abundance of soil microorganisms, among them, the impact of intercropping with 1.2% biochar was the most obvious. Biochar had a little effect on the bacterial community diversity but reduced the fungal community diversity. The community structures of soil bacteria and fungi were mainly affected by NO${}_{3}^{-}$-N, pH, EC, and moisture content. Besides, biochar and intercropping could increase some potentially beneficial organisms (e.g., *Pseudeurotium* and *Solirubrobacter*) and decrease potentially pathogenic organisms (e.g., *Kribbella* and *Ilyonectria*). The addition of biochar increased the connection between beneficial organisms, and the proportion of positive interactions of microbial community in the intercropping system. These results indicated that the suitable combination of biochar and tomato/potato–onion intercropping system was an effective way to improve soil health and crop productivity.

## Data Availability Statement

The original contributions presented in the study are publicly available. This data can be found here: National Center for Biotechnology Information (NCBI) BioProject database under accession number PRJNA657435.

## Author Contributions

XH: conceptualization, data curation, methodology, visualization, and writing – original draft. HX, DG, MR, and XZ: writing – review and editing. FW: project administration, supervision, and writing – review and editing. All authors contributed to the article and approved the submitted version.

## Conflict of Interest

The authors declare that the research was conducted in the absence of any commercial or financial relationships that could be construed as a potential conflict of interest.

## Publisher’s Note

All claims expressed in this article are solely those of the authors and do not necessarily represent those of their affiliated organizations, or those of the publisher, the editors and the reviewers. Any product that may be evaluated in this article, or claim that may be made by its manufacturer, is not guaranteed or endorsed by the publisher.
