# Supplementary material for: Biochar and Intercropping With Potato–Onion Enhanced the Growth and Yield Advantages of Tomato by Regulating the Soil Properties, Nutrient Uptake, and Soil Microbial Community
Source: Front Microbiol. 2021 Aug 27;12:695447. doi: 10.3389/fmicb.2021.695447 (PMC8429823; doi:10.3389/fmicb.2021.695447)
Supplement: Supplementary file 1 [file Data_Sheet_1.docx]

**Biochar and Intercropping With Potato–Onion Enhanced the Growth and Yield Advantages of Tomato by Regulating the Soil Properties, Nutrient Uptake, and Soil Microbial Community**

**
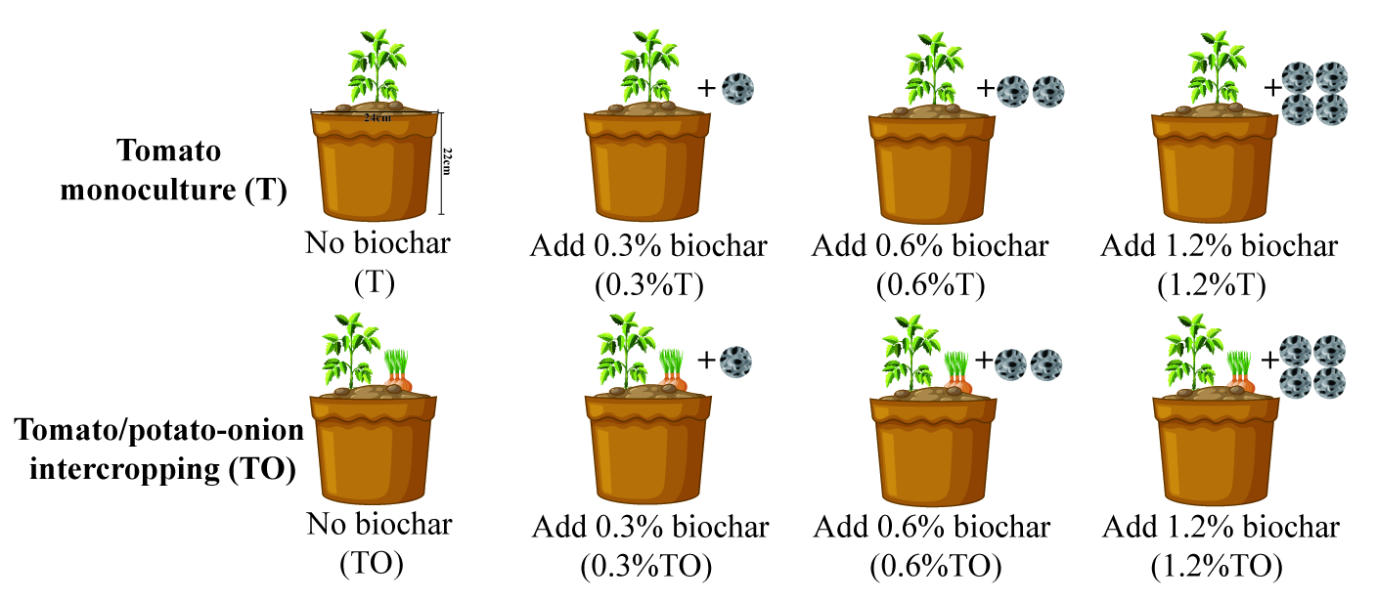
**

**Fig. S1** Schematic diagram of experimental design of two planting treatments with or without biochar.

**Table S1** Top 10 most abundant bacterial phylum of all soil samples.

| Phylum | 0%T | 0.3%T | 0.6%T | 1.2%T | 0%TO | 0.3%TO | 0.6%TO | 1.2%TO |
| --- | --- | --- | --- | --- | --- | --- | --- | --- |
| Proteobacteria | 28.65±1.99a | 29.85±4.52a | 33.09±6.26a | 28.46±2.63a | 29.53±5.30a | 26.45±3.79a | 30.22±2.10a | 27.53±1.86a |
| Actinobacteria | 19.45±1.39c | 18.76±0.88c | 21.10±4.33bc | 21.25±0.93bc | 23.73±1.68ab | 21.34±1.85bc | 25.55±2.18a | 23.85±1.45ab |
| Chloroflexi | 16.42±1.33a | 18.31±4.46a | 16.06±7.37a | 18.05±0.69a | 17.70±3.91a | 16.63±3.91a | 15.10±1.91a | 20.82±2.07a |
| Acidobacteria | 8.57±1.76a | 6.83±2.50a | 6.04±2.39a | 9.15±2.63a | 6.72±1.87a | 8.61±2.15a | 7.73±1.55a | 7.87±1.24a |
| Bacteroidetes | 6.37±1.83ab | 6.92±1.15a | 5.02±1.35abc | 4.43±0.67bc | 4.45±1.68bc | 5.22±1.35abc | 4.54±0.17bc | 3.67±0.14c |
| Gemmatimonadetes | 5.54±0.24a | 5.06±0.63a | 5.36±1.20a | 5.11±0.58a | 5.13±0.83a | 4.88±0.91a | 4.69±0.37a | 4.52±0.34a |
| Firmicutes | 4.95±0.23ab | 4.22±0.55bc | 4.15±0.76bc | 4.64±0.13abc | 4.13±0.72c | 5.31±0.40a | 4.18±0.26bc | 3.13±0.19d |
| Patescibacteria | 4.31±0.55abc | 4.66±0.22ab | 4.44±1.50abc | 4.09±0.12bc | 4.42±0.99abc | 5.44±0.92a | 3.39±0.26bc | 3.34±0.20c |
| Planctomycetes | 0.99±0.14ab | 1.02±0.30ab | 0.80±0.21b | 0.87±0.06b | 0.70±0.27b | 1.21±0.11a | 0.71±0.17b | 0.98±0.18ab |
| Cyanobacteria | 1.01±0.14a | 0.87±0.31a | 0.75±0.31a | 0.67±0.47a | 0.62±0.19a | 0.72±0.17a | 0.75±0.21a | 0.94±0.20a |

T and TO indicate tomato monoculture and intercropped with potato-onion, respectively. 0%, 0.3%, 0.6% and 1.2% indicate the biochar rates. Different letters indicate statistically significant differences among treatments (*P* < 0.05).

**Table S2** Top 5 most abundant fungal phylum of all soil samples.

| Phylum | 0%T | 0.3%T | 0.6%T | 1.2%T | 0%TO | 0.3%TO | 0.6%TO | 1.2%TO |
| --- | --- | --- | --- | --- | --- | --- | --- | --- |
| Ascomycota | 64.58±2.18a | 71.99±2.25a | 66.08±5.01a | 68.28±2.01a | 70.42±3.22a | 71.27±3.14a | 68.32±2.81a | 66.18±2.31a |
| Mortierellomycota | 27.18±2.08a | 23.03±1.47a | 26.22±3.43a | 24.46±0.44a | 22.73±2.51a | 22.23±2.20a | 24.27±3.00a | 25.97±1.54a |
| Basidiomycota | 6.55±0.29a | 4.08±0.82b | 5.13±0.18ab | 5.66±1.59ab | 5.51±0.83ab | 5.46±0.83ab | 6.04±0.86a | 6.04±0.64a |
| Olpidiomycota | 0.15±0.04a | 0.08±0.05b | 0.04±0.02b | 0.08±0.03b | 0.16±0.01a | 0.08±0.01b | 0.03±0.03b | 0.19±0.01a |
| Chytridiomycota | 0.08±0.03a | 0.00±0.00b | 0.02±0.01b | 0.01±0.01b | 0.01±0.01b | 0.02±0.00b | 0.02±0.01b | 0.01±0.00b |

T and TO indicate tomato monoculture and intercropped with potato-onion, respectively. 0%, 0.3%, 0.6% and 1.2% indicate the biochar rates. Different letters indicate statistically significant differences among treatments (*P* < 0.05).

**Table S3** Relative abundance (%) of the dominant bacterial (top 50) classfied genera of all soil samples.

| Genus | 0%T | 0.3%T | 0.6%T | 1.2%T | 0%TO | 0.3%TO | 0.6%TO | 1.2%TO | |
| --- | --- | --- | --- | --- | --- | --- | --- | --- | --- |
| *MND1* | 1.66±0.15a | 1.52±0.33a | 1.63±0.57a | 1.89±0.24a | 1.57±0.03a | 1.47±0.21a | 1.32±0.07a | 1.69±0.17a | |
| *Streptomyces* | 1.08±0.04d | 1.14±0.13cd | 1.31±0.13b | 1.04±0.05d | 1.34±0.06b | 1.29±0.08bc | 1.66±0.14a | 1.04±0.02d | |
| *Aeromicrobium* | 0.90±0.12b | 0.88±0.29b | 0.82±0.25b | 1.41±0.17a | 1.05±0.29ab | 1.22±0.37ab | 0.94±0.16b | 0.91±0.09b | |
| *Haliangium* | 1.03±0.05a | 0.91±0.19a | 0.94±0.27a | 0.98±0.12a | 1.03±0.09a | 0.87±0.10a | 1.00±0.01a | 1.07±0.04a | |
| *Gaiella* | 0.86±0.08bc | 0.72±0.15c | 0.97±0.64abc | 0.88±0.04bc | 1.16±0.22abc | 0.81±0.21c | 1.35±0.17a | 1.31±0.12ab | |
| *Clostridium_sensu_stricto_1* | 1.30±0.21a | 0.96±0.17bc | 0.81±0.17cd | 1.14±0.05abc | 0.82±0.24cd | 1.28±0.36ab | 0.56±0.04c | 0.58±0.08c | |
| *Pseudomonas* | 0.77±0.11bc | 0.59±0.03cde | 0.99±0.30b | 0.61±0.11cd | 0.42±0.17de | 0.41±0.09de | 3.08±0.20a | 0.32±0.02e | |
| *Rubrobacter* | 0.90±0.23b | 0.72±0.13b | 0.76±0.08b | 0.80±0.04b | 0.84±0.19b | 0.94±0.10ab | 1.02±0.08a | 0.76±0.11b | |
| *Arthrobacter* | 0.84±0.23a | 0.90±0.29a | 0.67±0.19a | 0.85±0.03a | 0.98±0.38a | 0.92±0.17a | 0.76±0.13a | 0.81±0.06a | |
| *Gemmatimonas* | 0.88±0.05abc | 0.60±0.10e | 0.81±0.20abcd | 0.92±0.08ab | 1.00±0.17a | 0.69±0.04cde | 0.63±0.10de | 0.79±0.07bcde | |
| *Devosia* | 0.63±0.10cd | 0.86±0.07ab | 0.92±0.08a | 0.74±0.06bc | 0.83±0.11ab | 0.68±0.09cd | 0.61±0.04cd | 0.56±0.02d | |
| *Nocardioides* | 0.71±0.09ab | 0.48±0.10c | 0.44±0.02c | 0.80±0.07ab | 0.86±0.22a | 0.63±0.16bc | 0.92±0.15a | 0.91±0.09a | |
| *Sphingomonas* | 0.82±0.05a | 0.72±0.16a | 0.82±0.16a | 0.61±0.11ab | 0.75±0.16a | 0.71±0.15a | 0.71±0.08a | 0.19±0.06b | |
| *Nitrospira* | 0.72±0.07ab | 0.72±0.14ab | 0.92±0.19a | 0.62±0.08bc | 0.66±0.16bc | 0.69±010bc | 0.49±0.05c | 0.48±0.10c | |
| *Chryseolinea* | 0.83±0.39b | 1.33±0.20a | 0.59±0.24bc | 0.40±0.08c | 0.65±0.33bc | 0.57±0.10bc | 0.54±0.02bc | 0.34±0.02c | |
| *Galbitalea* | 0.41±0.12d | 0.59±0.16bcd | 0.59±0.20bcd | 0.69±0.05abc | 0.79±0.15ab | 0.55±0.05cd | 0.70±0.17abc | 0.89±0.08a | |
| *Cellvibrio* | 0.36±0.05d | 0.62±0.15abc | 0.79±0.17a | 0.58±0.04bc | 0.58±0.15bc | 0.77±0.12a | 0.67±0.04ab | 0.46±0.03cd | |
| *Bacillus* | 0.60±0.01ab | 0.59±0.17ab | 0.70±0.16a | 0.54±0.05ab | 0.63±0.05ab | 0.64±0.09a | 0.65±0.03a | 0.47±0.03b | |
| *Blastococcus* | 0.42±0.03d | 0.37±0.03d | 0.56±0.35bcd | 0.50±0.05cd | 0.71±0.20abc | 0.46±0.10cd | 0.90±0.15a | 0.84±0.08ab | |
| *Microlunatus* | 0.61±0.06abc | 0.69±0.16ab | 0.77±0.28a | 0.47±0.02bcd | 0.57±0.14abc | 0.53±0.08bc | 0.39±0.02cd | 0.29±0.05d | |
| *Pedomicrobium* | 0.44±0.01cd | 0.49±0.11bcd | 0.63±0.09ab | 0.64±0.08ab | 0.69±0.13a | 0.46±0.06cd | 0.40±0.05d | 0.58±0.10abc | |
| *Steroidobacter* | 0.52±0.05ab | 0.77±0.19a | 0.56±0.16ab | 0.46±0.09b | 0.50±0.27b | 0.48±0.16b | 0.46±0.06b | | 0.34±0.07b |
| *Romboutsia* | 0.50±0.05abc | 0.44±0.08bcd | 0.40±0.08bcd | 0.58±0.04ab | 0.39±0.10bcd | 0.69±0.26a | 0.35±0.01cd | | 0.60±0.06ab |
| *Pseudonocardia* | 0.38±0.01bc | 0.30±0.05c | 0.44±0.21abc | 0.43±0.05bc | 0.52±0.10ab | 0.40±0.12bc | 0.61±0.06a | | 0.50±0.02ab |
| *Iamia* | 0.27±0.02c | 0.27±0.03c | 0.43±0.24bc | 0.35±0.04bc | 0.44±0.06b | 0.38±0.06bc | 0.66±0.02a | | 0.65±0.00a |
| *Acidibacter* | 0.44±0.01ab | 0.42±0.07ab | 0.51±0.16a | 0.46±0.15ab | 0.49±0.19ab | 0.33±0.11ab | 0.31±0.05b | | 0.50±0.05ab |
| *Actinomadura* | 0.31±0.04bc | 0.30±0.02c | 0.36±0.14bc | 0.28±0.05c | 0.41±0.02b | 0.33±0.05bc | 1.11±0.04a | | 0.34±0.04bc |
| *RB41* | 0.48±0.13a | 0.40±0.16a | 0.29±0.23a | 0.38±0.04a | 0.35±0.11a | 0.48±0.05a | 0.41±0.10a | | 0.35±0.04a |
| *Skermanella* | 0.40±0.01a | 0.40±0.11a | 0.43±0.06a | 0.39±0.01a | 0.39±0.10a | 0.43±0.03a | 0.25±0.03b | | 0.26±0.00b |
| *Agromyces* | 0.35±0.04ab | 0.29±0.05b | 0.37±0.13ab | 0.38±0.03ab | 0.40±0.04ab | 0.35±0.02ab | 0.41±0.09a | | 0.38±0.06ab |
| *Subgroup_10* | 0.30±0.02bc | 0.20±0.03c | 0.35±0.10bc | 0.37±0.05bc | 0.45±0.16ab | 0.30±0.15bc | 0.36±0.11bc | | 0.57±0.15a |
| *Bradyrhizobium* | 0.38±0.04ab | 0.37±0.07ab | 0.44±0.04a | 0.41±0.02a | 0.46±0.10a | 0.31±0.04bc | 0.25±0.02c | | 0.30±0.07bc |
| *Methyloceanibacter* | 0.32±0.04cd | 0.42±0.08ab | 0.45±0.08a | 0.33±0.04bcd | 0.38±0.05abc | 0.34±0.03bcd | 0.28±0.04cd | | 0.27±0.05d |
| *Amaricoccus* | 0.24±0.03de | 0.21±0.03e | 0.34±0.10bc | 0.27±0.03cde | 0.27±0.06cde | 0.33±0.06bcd | 0.48±0.06a | | 0.42±0.05ab |
| *Kribbella* | 0.29±0.01abc | 0.32±0.05ab | 0.39±0.11a | 0.32±0.02ab | 0.39±0.08a | 0.38±0.05a | 0.23±0.07bc | | 0.21±0.06c |
| *Solirubrobacter* | 0.20±0.04b | 0.21±0.03b | 0.26±0.15b | 0.24±0.02b | 0.34±0.16ab | 0.22±0.05b | 0.52±0.15a | | 0.47±0.13a |
| *Truepera* | 0.40±0.10a | 0.36±0.01abc | 0.22±0.09d | 0.22±0.03cd | 0.27±0.11abcd | 0.36±0.12abcd | 0.38±0.09ab | | 0.24±0.01bcd |
| *Hyphomicrobium* | 0.27±0.04bc | 0.36±0.09a | 0.34±0.04a | 0.32±0.05a | 0.36±0.10a | 0.27±0.02bc | 0.19±0.03c | | 0.29±0.07bc |
| *Lysobacter* | 0.28±0.04a | 0.24±0.04a | 0.31±0.12a | 0.31±0.06a | 0.32±0.08a | 0.30±0.08a | 0.35±0.04a | | 0.29±0.07a |
| *Microvirga* | 0.31±0.03ab | 0.26±0.04b | 0.36±0.06a | 0.31±0.04ab | 0.35±0.07a | 0.29±0.04ab | 0.26±0.04b | | 0.23±0.04b |
| *Bryobacter* | 0.33±0.04ab | 0.30±0.06bc | 0.41±0.10a | 0.29±0.03bc | 0.28±0.11bc | 0.26±0.06bc | 0.22±0.03c | | 0.25±0.01bc |
| *Bdellovibrio* | 0.27±0.03a | 0.22±0.04ab | 0.23±0.09ab | 0.28±0.06a | 0.21±0.06ab | 0.19±0.02ab | 0.17±0.02b | | 0.20±0.05ab |
| *Turicibacter* | 0.30±0.04a | 0.27±0.02a | 0.26±0.08a | 0.30±0.04a | 0.26±0.04a | 0.32±0.07a | 0.24±0.04ab | | 0.16±0.05b |
| *Bauldia* | 0.18±0.04b | 0.21±0.05b | 0.37±0.05a | 0.30±0.02a | 0.38±0.11a | 0.17±0.04b | 0.14±0.05b | | 0.31±0.03a |
| *Mesorhizobium* | 0.20±0.03c | 0.22±0.04bc | 0.27±0.03ab | 0.22±0.02bc | 0.29±0.06a | 0.24±0.03abc | 0.20±0.03c | | 0.20±0.03c |
| *Ilumatobacter* | 0.20±0.03bc | 0.19±0.04c | 0.26±0.06ab | 0.24±0.02abc | 0.29±0.05a | 0.20±0.03bc | 0.20±0.03bc | | 0.30±0.02a |
| *Flavobacterium* | 0.20±0.01bc | 0.30±0.06ab | 0.40±0.10a | 0.12±0.03c | 0.21±0.09bc | 0.24±0.07b | 0.20±0.05bc | | 0.10±0.04c |
| *Rhodanobacter* | 0.37±0.02a | 0.41±0.07a | 0.13±0.05c | 0.05±0.01d | 0.29±0.06b | 0.11±0.03cd | 0.34±0.01ab | | 0.07±0.02cd |
| *Ellin6067* | 0.27±0.03a | 0.22±0.04ab | 0.23±0.09ab | 0.28±0.06a | 0.21±0.06ab | 0.19±0.02ab | 0.17±0.02b | | 0.20±0.05ab |

T and TO indicate tomato monoculture and intercropped with potato-onion, respectively. 0%, 0.3%, 0.6% and 1.2% indicate the biochar rates. Different letters indicate statistically significant differences among treatments (*P* < 0.05).

**Table S4** Relative abundance (%) of the dominant fungi (top 50 classfeid) genera of all soil samples.

| Genus | 0%T | 0.3%T | 0.6%T | 1.2%T | 0%TO | 0.3%TO | 0.6%TO | 1.2%TO |
| --- | --- | --- | --- | --- | --- | --- | --- | --- |
| *Mortierella* | 27.15±2.09a | 22.81±1.43a | 26.09±3.45a | 24.31±0.42a | 22.61±2.52a | 21.99±2.15a | 24.19±2.98a | 25.76±1.55a |
| *Fusarium* | 5.12±1.12a | 6.28±0.48a | 5.29±1.31a | 6.61±0.55a | 6.02±1.19a | 4.74±0.10a | 4.74±0.40a | 4.34±0.71a |
| *Solicoccozyma* | 3.59±0.77a | 2.45±0.74a | 3.63±0.65a | 4.66±1.65a | 2.56±1.04a | 3.91±0.46a | 2.95±0.30a | 3.34±0.13a |
| *Pseudeurotium* | 1.46±0.41d | 2.16±0.34c | 2.97±0.29b | 2.02±0.11c | 2.85±0.16b | 1.32±0.21d | 2.00±0.42c | 4.89±0.35a |
| *Guehomyces* | 2.51±0.48a | 1.31±0.09b | 1.12±0.43b | 0.75±0.08b | 2.51±0.35a | 1.15±0.25b | 2.51±0.87a | 2.25±0.46a |
| *Schizothecium* | 1.01±0.17bc | 3.74±0.29a | 3.95±0.70a | 0.41±0.03d | 0.95±0.11bc | 1.11±0.09bc | 1.37±0.10bc | 0.78±0.13cd |
| *Acaulium* | 1.55±0.31ab | 1.41±0.13abc | 1.40±0.28bc | 1.32±0.07bc | 1.48±0.23ab | 1.47±0.36ab | 1.91±0.54a | 0.95±0.17c |
| *Cephaliophora* | 1.39±0.06b | 1.30±0.31b | 0.63±0.11d | 1.68±0.18b | 0.97±0.13cd | 1.48±0.20b | 2.22±0.56a | 1.24±0.15bc |
| *Cephalotrichum* | 0.63±0.11d | 0.62±0.11d | 1.12±0.11bc | 2.19±0.20a | 1.00±0.07bc | 0.50±0.02d | 0.88±0.13c | 1.13±0.23b |
| *Thelebolus* | 0.85±0.20ab | 0.66±0.12bc | 0.95±0.07a | 0.84±0.17ab | 0.77±0.10ab | 0.47±0.04c | 0.77±0.04ab | 0.66±0.07bc |
| *Aspergillus* | 0.76±0.22a | 0.81±0.19a | 0.77±0.20a | 0.40±0.06c | 0.64±0.17abc | 0.74±0.10ab | 0.76±0.18ab | 0.48±0.14bc |
| *Thermomyces* | 0.59±0.20abc | 0.72±0.06ab | 0.69±0.17ab | 0.68±0.05ab | 0.74±0.20a | 0.37±0.10c | 0.54±0.11abc | 0.46±0.25bc |
| *Humicola* | 0.56±0.12bc | 0.49±0.05c | 0.51±0.09c | 0.89±0.11a | 0.67±0.03b | 0.57±0.07bc | 0.45±0.04c | 0.50±0.12c |
| *Chaetomium* | 0.54±0.12bc | 0.61±0.03abc | 0.53±0.18bc | 0.73±0.12a | 0.70±0.12ab | 0.43±0.01c | 0.50±0.03c | 0.47±0.12c |
| *Plectosphaerella* | 0.27±0.04c | 0.27±0.04c | 0.37±0.06bc | 0.30±0.01c | 1.19±0.39a | 0.48±0.13bc | 0.63±0.25b | 0.96±0.11a |
| *Remersonia* | 0.52±0.11b | 0.84±0.11a | 0.59±0.11b | 0.51±0.19b | 0.46±0.10bc | 0.54±0.05b | 0.60±0.06b | 0.30±0.04c |
| *Wardomyces* | 0.46±0.13bcd | 0.35±0.05d | 0.44±0.06cd | 0.61±0.03ab | 0.52±0.15abc | 0.43±0.03cd | 0.64±0.13a | 0.37±0.07cd |
| *Aphanoascus* | 0.69±0.08a | 0.44±0.13bc | 0.33±0.04cd | 0.54±0.06b | 0.52±0.12b | 0.44±0.06bc | 0.27±0.02de | 0.19±0.07e |
| *Trichoderma* | 0.43±0.16bc | 0.15±0.02d | 0.60±0.25b | 0.57±0.19b | 0.95±0.23a | 0.06±0.01d | 0.16±0.06d | 0.19±0.12cd |
| *Gamsia* | 0.37±0.04ab | 0.33±0.03b | 0.42±0.04a | 0.40±0.06a | 0.33±0.04b | 0.26±0.03c | 0.33±0.01b | 0.33±0.01b |
| *Nectria* | 0.17±0.06e | 0.30±0.02cd | 0.34±0.01bc | 0.41±0.06b | 0.54±0.12a | 0.22±0.03de | 0.39±0.08bc | 0.30±0.03cd |
| *Fusicolla* | 0.11±0.01d | 0.85±0.05a | 0.26±0.04c | 0.25±0.05c | 0.54±0.10b | 0.19±0.02cd | 0.12±0.02d | 0.24±0.03c |
| *Mycothermus* | 0.16±0.03d | 0.45±0.09a | 0.34±0.08b | 0.25±0.02c | 0.18±0.02cd | 0.15±0.02d | 0.25±0.03c | 0.16±0.01d |
| *Preussia* | 0.23±0.01bc | 0.19±0.02c | 0.28±0.07ab | 0.20±0.02c | 0.19±0.03c | 0.32±0.07a | 0.24±0.04bc | 0.21±0.04bc |
| *Podospora* | 0.10±0.03d | 0.11±0.00d | 0.29±0.04bc | 0.25±0.05c | 0.39±0.09a | 0.10±0.03d | 0.11±0.01d | 0.35±0.06ab |
| *Gymnoascus* | 0.23±0.02b | 0.22±0.07bc | 0.21±0.07bc | 0.31±0.01a | 0.19±0.04bc | 0.19±0.02bc | 0.16±0.03cd | 0.10±0.02d |
| *Acremonium* | 0.21±0.03bc | 0.31±0.02a | 0.15±0.02d | 0.24±0.03b | 0.16±0.05cd | 0.23±0.05b | 0.16±0.01d | 0.12±0.02d |
| *Cylindrocarpon* | 0.10±0.01e | 0.18±0.04c | 0.13±0.03cde | 0.35±0.05b | 0.16±0.03cd | 0.13±0.03cde | 0.44±0.04a | 0.10±0.01de |
| *Penicillium* | 0.41±0.19a | 0.10±0.01b | 0.10±0.03b | 0.08±0.03b | 0.40±0.13a | 0.10±0.01b | 0.17±0.05b | 0.14±0.03b |
| *Stachybotrys* | 0.06±0.02e | 0.04±0.01e | 0.06±0.01e | 0.04±0.01e | 0.29±0.05b | 0.44±0.05a | 0.22±0.03c | 0.15±0.03d |
| *Pyrenochaeta* | 0.06±0.01cd | 0.11±0.01c | 0.04±0.01d | 0.08±0.01cd | 0.32±0.07a | 0.33±0.04a | 0.11±0.03c | 0.19±0.03b |
| *Tetracladium* | 0.21±0.03a | 0.18±0.03ab | 0.10±0.00c | 0.04±0.01d | 0.19±0.03ab | 0.10±0.00c | 0.16±0.05b | 0.06±0.01cd |
| *Talaromyces* | 0.13±0.03b | 0.14±0.02b | 0.12±0.02b | 0.07±0.02c | 0.19±0.03a | 0.15±0.03b | 0.15±0.02b | 0.08±0.01c |
| *Emericellopsis* | 0.08±0.03def | 0.19±0.04b | 0.09±0.02de | 0.15±0.02bc | 0.07±0.02ef | 0.12±0.02cd | 0.26±0.03a | 0.04±0.00f |
| *Phialophora* | 0.05±0.01c | 0.03±0.01c | 0.29±0.11a | 0.08±0.01bc | 0.14±0.02b | 0.02±0.01c | 0.10±0.05bc | 0.27±0.05a |
| *Cercophora* | 0.18±0.03b | 0.04±0.01ef | 0.05±0.01ef | 0.09±0.02cd | 0.12±0.03c | 0.02±0.01f | 0.07±0.02de | 0.38±0.01a |
| *Geminibasidium* | 0.11±0.03b | 0.12±0.04ab | 0.10±0.05b | 0.09±0.01b | 0.17±0.02ab | 0.11±0.04ab | 0.19±0.09a | 0.09±0.03b |
| *Dicyma* | 0.07±0.00d | 0.06±0.00de | 0.13±0.02bc | 0.21±0.03a | 0.12±0.01c | 0.02±0.01f | 0.04±0.01ef | 0.15±0.01b |
| *Sagenomella* | 0.11±0.02ab | 0.07±0.02c | 0.09±0.02bc | 0.14±0.02a | 0.11±0.02ab | 0.09±0.01bc | 0.11±0.03ab | 0.08±0.00bc |
| *Gibberella* | 0.08±0.02d | 0.09±0.02cd | 0.05±0.02d | 0.13±0.04ab | 0.09±0.01cd | 0.13±0.02abc | 0.15±0.03a | 0.09±0.01cd |
| *Olpidium* | 0.15±0.04a | 0.08±0.05b | 0.04±0.02b | 0.08±0.03b | 0.16±0.01a | 0.08±0.01b | 0.03±0.03b | 0.19±0.07a |
| *Arthrographis* | 0.13±0.05ab | 0.06±0.01c | 0.06±0.01c | 0.09±0.02bc | 0.10±0.03abc | 0.09±0.02bc | 0.14±0.01a | 0.10±0.01abc |
| *Pseudogymnoascus* | 0.10±0.08a | 0.07±0.03a | 0.08±0.07a | 0.11±0.05a | 0.15±0.14a | 0.08±0.06a | 0.12±0.08a | 0.05±0.04a |
| *Geomyces* | 0.08±0.07a | 0.05±0.01a | 0.06±0.04a | 0.14±0.05a | 0.11±0.10a | 0.10±0.08a | 0.07±0.06a | 0.04±0.03a |
| *Ilyonectria* | 0.13±0.00a | 0.08±0.02bc | 0.05±0.01c | 0.05±0.02c | 0.09±0.04b | 0.07±0.01bc | 0.06±0.01bc | 0.06±0.02bc |
| *Ampelomyces* | 0.03±0.01cd | 0.13±0.01b | 0.01±0.00d | 0.07±0.03c | 0.27±0.07a | 0.01±0.00d | 0.01±0.00d | 0.01±0.00d |
| *Holtermanniella* | 0.03±0.02cd | 0.03±0.01cd | 0.05±0.01bc | 0.01±0.00d | 0.04±0.01cd | 0.12±0.05a | 0.08±0.03ab | 0.10±0.03a |
| *Myrothecium* | 0.06±0.03bc | 0.04±0.01bc | 0.08±0.04ab | 0.03±0.01c | 0.04±0.01bc | 0.03±0.01c | 0.11±0.04a | 0.04±0.01bc |
| *Zopfiella* | 0.06±0.01ab | 0.06±0.03ab | 0.09±0.04a | 0.06±0.02ab | 0.02±0.02c | 0.01±0.01c | 0.03±0.02bc | 0.08±0.03a |
| *Wallemia* | 0.04±0.03b | 0.03±0.00b | 0.04±0.02b | 0.03±0.02b | 0.03±0.01b | 0.07±0.04ab | 0.10±0.04a | 0.04±0.01b |

T and TO indicate tomato monoculture and intercropped with potato-onion, respectively. 0%, 0.3%, 0.6% and 1.2% indicate the biochar rates. Different letters indicate statistically significant differences among treatments (*P* < 0.05).

**Table S5** The co-occurrence network analysis topological properties of soil microbial communities.

| Network indicators | 0%T | 0.3%T | 0.6%T | 1.2%T | 0%TO | 0.3%TO | 0.6%TO | 1.2%TO |
| --- | --- | --- | --- | --- | --- | --- | --- | --- |
| Nodes | 75 | 72 | 70 | 68 | 83 | 71 | 77 | 65 |
| Edges | 1003 | 1037 | 913 | 888 | 1212 | 1029 | 1075 | 722 |
| Positive correlation edge numbers | 546  (56.23%) | 758  (73.10%) | 531  (58.16%) | 482  (54.28%) | 640  (52..81%) | 661  (64.24%) | 634  (58.98%) | 489  (67.73%) |
| Negative correlation edge numbers | 457  (43.77%) | 279  (26.90%) | 382  (41.84%) | 406  (45.72%) | 572  (47.19%) | 368  (35.76%) | 441  (41.02%) | 286  (32.27%) |
| Graph density (GD) | 0.361 | 0.406 | 0.378 | 0.390 | 0.356 | 0.414 | 0.367 | 0.371 |
| Modularity (MD) | 1.861 | 0.717 | 1.971 | 2.250 | 1.812 | 1.220 | 1.489 | 1.146 |
| Clustering coefficient (CC) | 0.988 | 0.988 | 0.987 | 0.986 | 0.989 | 0.987 | 0.989 | 0.985 |

T and TO indicate tomato monoculture and intercropped with potato-onion, respectively. 0%, 0.3%, 0.6%, and 1.2% indicate the biochar rates.
